# Non-fasting Changes in Blood Lipids After Three Daily Meals Within a Day in Chinese Inpatients With Cardiovascular Diseases

**DOI:** 10.3389/fcvm.2022.799300

**Published:** 2022-04-12

**Authors:** Yangrong Tan, Qiuzhen Lin, Jin Xu, Liyuan Zhu, Liling Guo, Yingying Xie, Xiao Du, Shilan Zhang, Tie Wen, Ling Liu

**Affiliations:** ^1^Department of Cardiovascular Medicine, The Second Xiangya Hospital, Central South University, Changsha, China; ^2^Research Institute of Blood Lipid and Atherosclerosis, Central South University, Changsha, China; ^3^Modern Cardiovascular Disease Clinical Technology Research Center of Hunan Province, Changsha, China; ^4^Cardiovascular Disease Research Center of Hunan Province, Changsha, China; ^5^Department of Emergency Medicine, The Second Xiangya Hospital, Central South University, Changsha, China; ^6^Emergency Medicine and Difficult Diseases Institute, The Second Xiangya Hospital, Central South University, Changsha, China

**Keywords:** non-fasting, blood lipids, three daily meals, Chinese inpatients, cardiovascular diseases

## Abstract

**Background:**

Non-fasting (i.e., postprandial) lipid detection is recommended in clinical practice. However, the change in blood lipids in Chinese patients with cardiovascular diseases after three daily meals has never been reported yet.

**Methods:**

Serum levels of blood lipids were measured or calculated in 77 inpatients (48 men and 29 women) at high or very high risk of atherosclerotic cardiovascular disease (ASCVD) in the fasting state and at 4 h after three meals within a day according to their diet habits.

**Results:**

Female patients showed significantly higher level of high-density lipoprotein cholesterol (HDL-C) than male patients, and the gender difference in other lipid parameters did not reach statistical significance at any time-point. Levels of triglyceride (TG) and remnant cholesterol (RC) increased, while that of low-density lipoprotein cholesterol (LDL-C) decreased significantly after three meals (*p* < 0.05). Levels of HDL-C, total cholesterol (TC), and non-high-density lipoprotein cholesterol (non-HDL-C) showed smaller changes after three meals. Percent reductions in the non-fasting LDL-C levels after lunch and supper were around 20%, which were greater than that after breakfast. The percent reductions in the non-fasting non-HDL-C levels after three meals were smaller than those in the non-fasting LDL-C levels. Patients with TG level ≥ 2.0 mmol/L (177 mg/dL) after lunch had significantly greater absolute reduction of LDL-C level than those with TG level < 2.0 mmol/L (177 mg/dL) after lunch [–0.69 mmol/L (–27 mg/dL) vs. –0.36 mmol/L (–14 mg/dL), *p*<0.01]. There was a significant and negative correlation between absolute change in LDL-C level and that in TG level (*r* = −0.32) or RC level (*r* = −0.67) after lunch (both *p*<0.01).

**Conclusion:**

LDL-C level decreased significantly after three daily meals in Chinese patients at high or very high risk of ASCVD, especially when TG level reached its peak after lunch. Relatively, non-HDL-C level was more stable than LDL-C level postprandially. Therefore, when LDL-C level was measured in the non-fasting state, non-HDL-C level could be evaluated simultaneously to reduce the interference of related factors, such as postprandial hypertriglyceridemia, on detection.

## Introduction

Dyslipidemia is not only a risk factor of coronary heart disease (CHD) (1), but also accompanied by other risk factors, such as hypertension (2), diabetes (3), and overweight or obesity (4–6). Therefore, it is important to monitor blood lipids in both primary and secondary prevention of CHD. Previously, keeping fast was required before blood lipid test (7–9). However, it may cause discomfort of hungry and even the risk of hypoglycemia for patients with diabetes, the weak and elder subjects. Several studies with large population supported that non-fasting (i.e., postprandial) blood lipids had prognostic value for predicting myocardial infarction, ischemic heart disease, all-cause mortality, and cardiovascular mortality similar to fasting blood lipids, which include low-density cholesterol (LDL-C) and triglyceride (TG) (10–13). Then, a joint consensus statement was released in Europe, 2016, which recommends that non-fasting blood lipids can be tested routinely in clinical practice (14).

Cholesterol control requires both LDL-C and non-high-density lipoprotein cholesterol (non-HDL-C) levels reaching their goals, respectively. Non-HDL-C covers the cholesterol content in all atherogenic lipoproteins in the circulation. Remnant cholesterol (RC) is an important component of non-HDL-C, its level will elevate especially when TG level increases (15). RC means the cholesterol within hydrolyzed remnants of triglyceride-rich lipoproteins (TRLs). Those remnants can enter the subendothelial region like LDL and promote the formation and development of atherosclerosis. RC level can be estimated by a formula in both fasting and non-fasting states (14). Elevated non-fasting TG and RC levels were regarded as pathogenic risk factors for myocardial infarction (16). We previously found that TG and RC levels increased while LDL-C level decreased most prominently at 4 h after a daily breakfast in Chinese subjects, which includes those with CHD, overweight, and hypertension (17–19). Although the non-fasting changes in blood lipids were considered insignificant in Danish studies with large population (14), reduction in LDL-C level after a breakfast was found more obvious in several previous studies with Chinese subjects (17–19). The potential causes for the difference are still unclear.

Although the non-fasting variation in blood lipids after three daily meals had been explored in 41 healthy Chinese (20), the change in blood lipids in Chinese patients with cardiovascular diseases after three daily meals had never been reported yet. For patients at high or very high risk of atherosclerotic cardiovascular disease (ASCVD), monitoring blood lipids is extremely important, even in the non-fasting state. It is a fact in China that patients could visit their doctor for illness at any time of a day, perhaps in the fasting state, after breakfast, lunch, or even supper. A considerable number of outpatients with heterogenous conditions visited their doctors in the non-fasting state. While they came and left the hospital in a hurry, it was impossible to observe their changes in blood lipids after three meals within a day in the outpatient department. Therefore, the inpatients with similar ASCVD risk and heterogenous conditions in the wards of department of cardiovascular medicine become an eligible substitute for those outpatients. We aimed to observe the non-fasting changes in blood lipids in Chinese inpatients with cardiovascular diseases at high or very high risk of ASCVD after three daily meals within a day and explore the relationship between the non-fasting changes in LDL-C level and those in other lipid parameters.

## Subjects and Methods

### Subjects

A number of seventy-seven inpatients with cardiovascular diseases of Han Chinese in the Department of Cardiovascular Medicine of the Second Xiangya Hospital, Central South University were recruited in this study. There were 48 men (the male group) and 29 women (the female group). Inclusion criteria were as follows: (1) at high risk or very high risk of ASCVD, (2) those had returned to a daily diet according to their habits after the condition stabilized. According to “China cholesterol education program (CCEP) expert advice for the management of dyslipidemias to reduce cardiovascular risk (2019)” (21), patients at very high risk of ASCVD were defined as those with ASCVD, with diabetes mellitus (DM) and hypertension (HBP), or with DM and another risk factor and fasting LDL-C ≥ 2.6 mmol/L (100 mg/dL). Patients at high risk were defined as those with DM, HBP, and other two risk factors and fasting LDL-C ≥ 2.6 mmol/L (100 mg/dL), with chronic kidney disease stage 3 or 4, or with fasting LDL-C ≥ 4.9 mmol/L (190 mg/dL). Risk factors included age ≥ 45 years (men) or 55 years (women), smoking, body mass index (BMI) ≥ 28 Kg/m^2^, family history of premature ischemic cardiovascular disease, or low high-density lipoprotein cholesterol (HDL-C) level (i.e., HDL-C < 1.0 mmol/L or 39 mg/dL).

Coronary heart disease is defined as a history of myocardial infarction and/or angiographically proven coronary atherosclerosis in patients with angina pectoris. HBP is defined as systolic blood pressure (SBP) ≥ 140 mmHg or diastolic blood pressure (DBP) ≥ 90 mmHg, or a history of HBP (22). Obesity is defined as BMI ≥ 28 Kg/m^2^ according to “An expert consensus on the prevention and control of adult obesity in China (2011)” (23). Type 2 DM is diagnosed according to American Diabetes Association Standards of Medical Care in Diabetes (2020) (24).

Excluding criteria were as follows: (1) heart function stages III–IV class of New York Heart Association, (2) other endocrine diseases except for DM, (3) digestive diseases, (4) chronic wasting diseases and malignant tumor, (5) taking other hypolipidemic agents except for statins, and (6) acute or subacute myocardial infarction and ischemic stroke within 1 month considering the corresponding changes in stress response in blood lipids.

The study was approved by the Ethics Committee of the Second Xiangya Hospital of Central South University and confirmed to the 1975 Declaration of Helsinki. Informed consent was gained from all subjects prior to participation.

### Sample Collection

All patients took three meals, which include breakfast, lunch, and supper, according to their daily diet habits within one day after 12-h overnight fast. Meals were purchased from the hospital canteen. We previously found that the levels of TG, LDL-C, and RC changed most prominently at 4 h than at 2 h after a daily meal (17–19). Thus, venous blood samples were collected before breakfast and at 4 h after each meal. About 5 ml of blood sample was drawn from each subject at each time-point.

### Determination of Blood Lipid Levels

Serum was separated at 4^°^C. Serum levels of total cholesterol (TC) and TG levels were measured by the enzyme method, LDL-C and HDL-C levels were measured by the direct method (Wako, Japan) on a HITACHI 7170A analyzer (Instrument Hitachi Ltd., Tokyo, Japan) by a laboratory specialist who was blinded to this study. Non-HDL-C and RC levels were calculated according to two formulas, non-HDL-C = TC – HDL-C and RC = TC – HDL-C – LDL-C, respectively.

### Statistical Analyses

Data were analyzed with SPSS (version 25.0) and GraphPad Prism (version 8.0) software. Quantitative variables were shown as mean ± standard deviation (SD) unless otherwise noted, and qualitative data as numbers and percentages. Absolute changes in blood lipid levels were calculated by the formula, that is, each non-fasting level minus its fasting level. The percent changes in blood lipid levels were calculated by the formula, that is (each non-fasting level – fasting level) × 100%/fasting level. Normality tests for the lipid data were performed using the Shapiro–Wilk method. Difference between the intra- and intergroup means was analyzed by one-way ANOVA or *t*-test. Categorical variables were compared using chi-squared statistic tests. Correlations between lipid parameters were analyzed using a Spearman’s correlation analysis. All *p*-values were two-tailed, and *p* < 0.05 was considered statistically significant.

## Results

### Clinical Characteristics and the Fasting Blood Lipid Levels of the Two Groups

Among all patients, 35 were diagnosed as CHD, 28 were diagnosed as HBP, 30 were diagnosed as obesity, 11 were diagnosed as type 2 DM, 28 were diagnosed as cardiac arrhythmia, 4 were diagnosed as congenital heart defect, and 1 was diagnosed as alcoholic cardiomyopathy. As a whole, there were 37 at very high risk of ASCVD and the other 40 were at high risk of ASCVD.

There was no significant difference in age, BMI, the percentage of obesity, HBP, DM, CHD, or statin user between the male group and the female group. Moreover, there was no significant difference in the fasting level of TC, non-HDL-C, LDL-C, TG, or RC between the two groups. However, the percentage of smokers was significantly higher (*p* < 0.01) whereas the fasting HDL-C level was significantly lower in the male group (*p* < 0.05) (Table 1).

**TABLE 1:** Clinical characteristics and fasting blood lipid levels of the two groups.

	Male (*n* = 48)	Female (*n* = 29)
Age (year)	53.9 ± 11.8	58.2 ± 11.5
Body mass index (Kg/m^2^)	25.2 ± 3.1	23.9 ± 4.1
Obesity, *n* (%)	22 (45.8)	8 (27.6)
HBP, *n* (%)	18 (37.5)	10 (34.5)
DM, *n* (%)	7 (14.6)	4 (13.8)
CHD, *n* (%)	26 (54.2)	9 (31.0)
Current smoking, *n* (%)	18 (37.5)**	1 (3.4)
Statin user, *n* (%)	18 (37.5)	10 (34.5)
TC (mmol/L)	4.03 ± 0.85	4.06 ± 1.00
HDL-C (mmol/L)	1.00 ± 0.22*	1.20 ± 0.06
Non-HDL-C (mmol/L)	3.02 ± 0.82	2.90 ± 0.93
LDL-C (mmol/L)	2.56 ± 0.71	2.49 ± 0.83
TG (mmol/L)	1.84 ± 1.21	1.42 ± 0.80
RC (mmol/L)	0.46 ± 1.99	0.41 ± 0.26

*p < 0.05,

**p < 0.01 when compared to the female group. Cholesterol: 1 mmol/L = 38.7 mg/dL. TG: 1 mmol/L = 88.6 mg/dL.

### Non-fasting Blood Lipid Levels After Three Meals in the Two Groups

Only the difference in HDL-C level at 4 h after each meal between the female group and the male group reached statistical significance, however, that in TC, non-HDL-C, LDL-C, TG, or RC level between the two groups did not reach statistical significance.

The change trends of TC and non-HDL-C levels after three meals were similar. TC and non-HDL-C levels decreased at 4 h after breakfast, slightly recovered at 4 h after lunch, and further decreased at 4 h after supper. TC and non-HDL-C levels after supper were significantly lower than those in the fasting state, after breakfast and lunch, respectively (*p* < 0.05), except that the difference in TC level between in the fasting state and after supper did not reach statistical significance in the female group.

High-density lipoprotein cholesterol level was stable before and after meals. However, the female group had significant higher HDL-C level at 4 h after each meal than the male group (*p* < 0.05).

Low-density lipoprotein cholesterol level began to decrease at 4 h after breakfast, further dropped at 4 h after lunch, then maintained low at 4 h after supper in each group. The non-fasting LDL-C level after each meal was significantly lower than the fasting LDL-C level (*p* < 0.05). Additionally, LDL-C level after lunch was significantly lower than that after breakfast in each group (*p* < 0.05).

**FIGURE 1:**
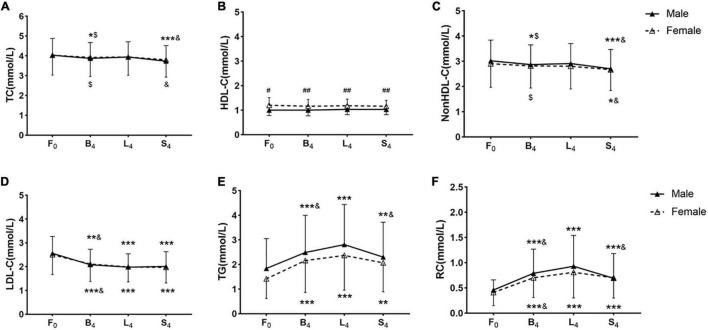
Non-fasting blood lipid levels after three meals in the two groups. **(A–F)** Non-fasting changes in levels of TC **(A)**, HDL-C **(B)**, non-HDL-C **(C)**, LDL-C **(D)**, TG **(E)**, and RC **(F)**. Levels of non-HDL-C and RC were calculated and estimated, respectively. F_0:_ at fasting state, B_4_: at 4 h after breakfast, L_4_: at 4 h after lunch, S_4_: at 4 h after supper. Cholesterol: 1 mmol/L = 38.7 mg/dL. TG: 1 mmol/L = 88.6 mg/dL. * *p* < 0.05, ** *p* < 0.01, and *** *p* < 0.001 when compared to the fasting level in the same group. ^$^*p* < 0.05 when compared to the level at 4 h after supper in the same group. ^&^*p* < 0.05 when compared to the level at 4 h after lunch in the same group. ^#^*p* < 0.05, ^##^*p* < 0.01 when compared to the female group at the same time-point.

Triglyceride level significantly increased at 4 h after breakfast, further elevated and reached the peak at 4 h after lunch, and then slightly dropped at 4 h after supper. The change in RC level after three meals was similar to that in TG level, but to a lesser extent. The difference in TG and RC levels between the fasting and non-fasting states reached statistical significance in both groups (*p* < 0.05). The male group had higher non-fasting TG and RC levels than the female group, but the difference did not reach statistical significance. TG and RC levels after lunch in the male group were significantly higher than those after breakfast and supper, respectively (*p* < 0.05). RC level after lunch in the female group was significantly higher than that after breakfast (*p* < 0.05) (Figure 1).

### Percent Changes in the Non-fasting Blood Lipid Levels in the Two Groups

**FIGURE 2:**
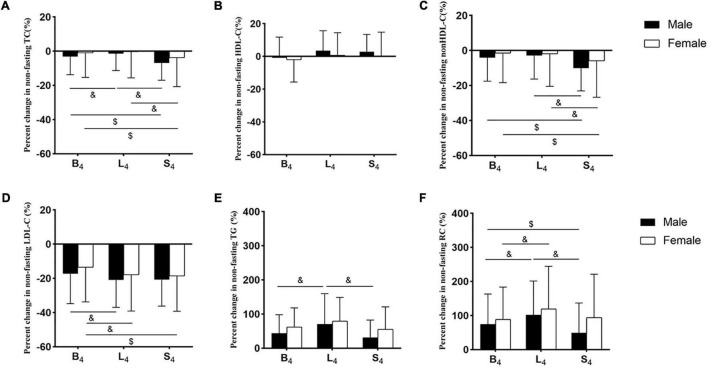
Percent changes in non-fasting blood lipid levels in the two groups. **(A–F)** Percent changes in non-fasting levels of TC **(A)**, HDL-C **(B)**, non-HDL-C **(C)**, LDL-C **(D)**, TG **(E**), and RC **(F)**. Levels of non-HDL-C and RC were calculated and estimated, respectively. The percent changes in blood lipid levels were calculated by the formula, that is (each non-fasting level - fasting level) × 100%/fasting level. B_4_: at 4 h after breakfast, L_4_: at 4 h after lunch, S_4_: at 4 h after supper. ^$^*p* < 0.05 when compared to the level at 4 h after supper in the same group. ^&^*p* < 0.05 when compared to the level at 4 h after lunch in the same group.

The difference in percent changes in the non-fasting lipids between the two groups at each non-fasting time-point did not reach statistical significance. Percent changes in the non-fasting levels of HDL-C, TC, and non-HDL-C in both groups were small (Figure 2). The percent change in HDL-C level was the smallest, and its maximum value among all non-fasting time-points was only 3.5%. The largest percent reductions in the non-fasting levels of TC and non-HDL-C after three meals were found after supper and reached 6.9 and 10.1%, respectively, and the largest percent reductions in the non-fasting LDL-C levels after three meals were around 20%. Percent changes in the non-fasting TG and RC levels were very prominent, which reaches the peak after lunch in both groups, and their largest values were more than 70 and 100%, respectively (Supplementary Table 1).

Percent reductions in TC and non-HDL-C levels after supper were significantly greater than those after breakfast and lunch, respectively, in each group (*p* < 0.05). Percent reduction of LDL-C level after lunch was significantly greater than that after breakfast in each group (*p* < 0.05). Percent changes in TG and RC levels after lunch in the male group were significantly greater than those after breakfast and supper, respectively (*p* < 0.05). Percent change in RC level after lunch in the female group was significantly greater than that after breakfast (*p* < 0.05) (Figure 2).

Absolute changes in the non-fasting blood lipid levels in the two groups are shown in Supplementary Table 1. The difference in absolute changes in the non-fasting lipids between both groups did not reach statistical significance. Absolute change in the non-fasting level of HDL-C in the two groups was smallest, followed by those of TC and non-HDL-C. The largest absolute reduction of the non-fasting LDL-C level was 0.58 mmol/L (22 mg/dL) after lunch. Absolute changes in the non-fasting TG and RC levels reached the peak after lunch. The largest absolute increases in the non-fasting TG and RC levels were 0.97 mmol/L (86 mg/dL) and 0.47 mmol/L (18 mg/dL), respectively (Supplementary Table 1).

### Effect of Statin Treatment on Fasting and Non-fasting Blood Lipids

There were 28 patients accepting statin treatment before admission. All subjects were divided into two subgroups according to accepting statin treatment (the statin subgroup, *n* = 28) or not (the non-statin subgroup, *n* = 49). There was no significant difference in the fasting or non-fasting level of TG, RC, or HDL-C between the two subgroups (data were not shown). The fasting and non-fasting levels of TC, LDL-C, and non-HDL-C in the statin subgroup were significantly lower than those in the non-statin subgroup (*p* < 0.05). However, the difference in the non-fasting changes in TC, LDL-C, and non-HDL-C levels after each meal between the two subgroups did not reach statistical significance (data were not shown).

### Correlation Analysis

Taking all subjects as a whole, correlation analysis showed that there was a significant and negative correlation between absolute change in LDL-C level (ΔLDL-C) and that in TG level (ΔTG) at 4 h after breakfast (*r* = −0.25, *p*< 0.05) or lunch (*r* = −0.32, *p*< 0.01). Moreover, there were significant and negative relationships between ΔLDL-C and that in RC level (ΔRC) at 4 h after three meals (breakfast: *r* = −0.58; lunch: *r* = −0.67; supper: *r* = −0.49, all *p*< 0.001) (Table 2).

**TABLE 2:** Correlation coefficients between absolute change in LDL-C level (mmol/L) and those in TG and RC levels (mmol/L) after each meal.

*r*	Δ TG_*B*4_	Δ TG_*L*4_	Δ TG_*S*4_	Δ RLP-C_*B*4_	Δ RLP-C_*L*4_	Δ RC_*S*4_
ΔLDL-C_*B*4_	–0.25			–0.58		
ΔLDL-C_*L*4_		–0.32			–0.67	
ΔLDL-C_*S*4_			–0.19			–0.49
*P*	0.028	0.004	0.103	0.000	0.000	0.000

*Absolute change in LDL-C level (ΔLDL-C), TG level (ΔTG), or RC level (ΔRC) after each meal. Absolute changes in blood lipid levels were calculated by the formula, that is, each non-fasting level minus fasting level. B_4_: at 4 h after breakfast, L_4_: at 4 h after lunch, S_4_: at 4 h after supper. Cholesterol: 1 mmol/L = 38.7 mg/dL. TG: 1 mmol/L = 88.6 mg/dL.*

**FIGURE 3:**
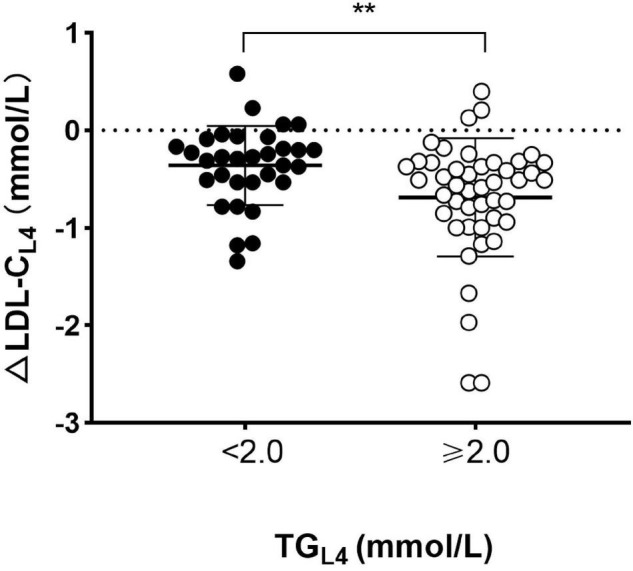
Comparison of absolute reduction in LDL-C level between patients with and without high TG at 4 h after lunch. Absolute changes in LDL-C level (ΔLDL-CL_4_) and TG level (ΔTGL_4_) at 4 h after lunch. Solid circles represented patients with high TG (TG ≥ 2.0 mmol/L) and open circles represented those without high TG (TG < 2.0 mmol/L) at 4 h after lunch. Cholesterol: 1 mmol/L = 38.7 mg/dL. TG: 1 mmol/L = 88.6 mg/dL. **p < 0.01 when compared to each other.

The European joint consensus statement recommended that non-fasting TG level after a daily meal should be less than 2.0 mmol/L (177 mg/dL) (14). For that, TG level reached the peak after lunch, and then, all subjects were divided into two parts according to TG level at 4 h after lunch (TG_*L*4_). Subjects with TG_*L*4_ ≥ 2.0 mmol/L (177 mg/dL) had significantly greater ΔLDL-C than those with TG_*L*4_ < 2.0 mmol/L (177 mg/dL) [−0.69 mmol/L (−27 mg/dL) vs. −0.36 mmol/L (−14 mg/dL), *p* s< 0.01] (Figure 3).

## Discussion

This study was the first one to explore the non-fasting blood lipids in Chinese patients at high or very high risk of ASCVD after three daily meals within a day. They showed greater TG elevation and LDL-C reduction after three meals than those healthy Chinese in another study (20). HDL-C level was stable postprandially in this study. TC and non-HDL-C levels reached their lowest levels after supper. Interestingly, the greatest differences in changes of LDL-C as well as TG and RC levels were seen after lunch but not after supper. There were significant and negative correlations between absolute change in LDL-C level and those in TG and RC levels, especially after lunch. It indicated that high TG after lunch could exert a potential role in LDL-C reduction. The findings could be helpful to guide the non-fasting blood lipids detection of those with similar ASCVD risk, including the outpatients.

We noticed that the greatest differences in changes of blood lipids, such as TG and RC elevations, were seen after lunch but not after supper. It was different from TG changes during a day in the European subjects (25) and could be related to Chinese diet habits. During a day, Chinese lunch usually is more formal and contains higher calories than breakfast, which makes TG level rises further based on the elevation after breakfast. Conversely, people would consciously control the calories of supper according to the view of traditional Chinese health preservation. There is a Chinese saying that filling an empty stomach to 70% fullness is enough for supper. Thus, TG reached its peak level after lunch during a day.

Hypertriglyceridemia is often accompanied by low HDL-C level. Cholesteryl ester transfer protein (CETP) is in charge of the redistribution of cholesteryl ester and TG between lipoproteins in plasma (26–29). CETP can transfer cholesteryl ester from LDL and HDL to TRLs and their remnants and return TG back to LDL and HDL (30–32). It was found that gender and CETP gene polymorphism were both associated with the pathological distribution of TG levels after oral fat tolerance test in heterozygotes for familial hypercholesterolemia (*n* = 80). Specifically, men carrying the B2 allele of CETP polymorphism showed a higher postprandial TG peak and a delayed return to basal levels compared with women carrying B2 (33). The male group showed higher TG while lower HDL-C levels than the female group in this study; however, the difference in TG levels after daily meals between groups did not reach statistical significance. It may be due to the different underlying diseases and diets between the two studies.

The transfer of cholesterol between lipoproteins may result in low LDL-C level in the situation of non-fasting hypertriglyceridemia. It was found that patients with hypertriglyceridemia showed greater net cholesteryl ester transfer, which leads to enhanced accumulation of cholesteryl ester in TRLs and their remnants, than normolipidemic individuals (34). The increase in TG content will facilitate the hydrolysis of TG of LDL by lipoprotein lipase and produce more small and dense LDL (smLDL), which could induce low LDL-C level (30, 34, 35). When TG level is optimal (<1.2 mmol/L or 106 mg/dL), there is normal size LDL in plasma. Under moderate-severe hypertriglyceridemia (TG: 1.7–5.7 mmol/L or 150–505 mg/dL), there are increased both TRLs remnants and smLDL particles in plasma (36). Under the same mechanism, hypertriglyceridemia could promote the clearance of HDL, which results in the non-fasting reduction of HDL-C level. Strangely, HDL-C level was very stable after three daily meals, which suggested that there could be other explanations for the non-fasting reduction of LDL-C level in this study.

Japanese scholars reported that LDL-C levels determined by a homogeneous assay significantly decreased from after breakfast until midnight and then returned to the baseline level in the next morning (37). They thought that the circadian change in LDL-C level might be related to a combination of multiple factors, which includes reduced cholesterol synthesis during the day while enhanced cholesterol synthesis at night. It was found that cholesterol synthesis, measured as serum lathosterol level, decreased from breakfast and reached its lowest level at about 6 p.m and then gradually increased until the next morning. In contrast to the marked diurnal change in cholesterol metabolism, LDL-C level still remained stable during the diurnal cycle in the European subjects (25). Notably, the greatest reduction of LDL-C first appeared after lunch but not after supper in the male group, when there were non-fasting TG and RC peaks. It makes us to consider the potential impact of postprandal hypertriglyceridemia on LDL-C detection, in addition to other factors.

Recently, new evidence showed that the non-fasting reduction of LDL-C level determined enzymatically was not confirmed by nuclear magnetic resonance (NMR)-based method. There was no significant reduction in both LDL particle and its cholesterol content determined by NMR. Only the non-fasting large LDL particles and their cholesterol content were significantly decreased (38). Moreover, there was significant increase of cholesterol in medium LDL particles. The redistribution of cholesterol in LDL subclasses after a daily meal could lead to the non-fasting reduction in LDL-C level. It indicates that the reduction in LDL-C level in the non-fasting state may be due to limitations of detection kits being more sensitive to large particles than to medium and small particles, two of which are predominant in patients with ASCVD. Therefore, the defect that some commercial kits can only detect cholesterol in part of LDL particles may be one reason for the non-fasting reduction in LDL-C level, especially under the condition of hypertriglyceridemia (38). Moreover, the non-fasting reduction in LDL-C level needs to be explained more cautiously under this condition.

In addition, Japanese scholars found that the reduction in LDL-C level from the baseline was significantly greater in the statin subgroup than in the non-statin subgroup after breakfast and before lunch. They speculated that LDL-C reduction during the day was partly due to the enhanced clearance of LDL from the circulation, which derives from the inhibition of liver cholesterol synthesis by statins (37). However, we did not observe a similar phenomenon, although there was a relatively larger sample size in this study. We noted that subjects in the Japanese study took standard meals other than daily meals, which could lead to the difference in this aspect between the two studies.

It is worth noting that measurement of either a fasting or non-fasting lipid profile is effective in estimating cardiovascular risk (39) and for documenting a baseline LDL-C level to evaluate cholesterol control in patients with CHD (40). In other words, the non-fasting reduction in LDL-C level either obvious or not in Chinese subjects should not be a factor interfering with the application of non-fasting blood lipid detection in clinical practice. In most primary hospitals in China, more sensitive NMR is unavailable or too expensive for lipid test. When cheaper commercial kits are used to detect blood lipids, is there any valuable lipid parameters more stable than LDL-C in the non-fasting state?

In the abovementioned studies with Japanese (37) and European subjects (25), the change in non-HDL-C level was not mentioned. The change in non-HDL-C level was similar to that in TC level after three meals and relatively small if considering its percent changes when compared to that in LDL-C level, especially after breakfast and lunch. It supported that it is feasible to detect the non-fasting non-HDL-C level in Chinese patients at high or very high risk of ASCVD during the day. Recently, it was reported that non-HDL-C level was more stable than LDL-C level in assessing the percent attainment of the non-fasting lipids for Chinese patients with CHD (40). It indicated that non-HDL-C level could be quite important, either in the fasting or non-fasting state, especially when considering it nearly covers the cholesterol of all atherosclerotic lipoproteins in the circulation. This view should be focused by more clinicians.

There were several limitations in this study. First, the sample size was relatively small, and the aggregation of subjects with different pathophysiological conditions could render data interpretation complicated. Second, lipid profile in the next morning was not measured to assess whether the lipid profile returned to baseline after overnight fast. Third, it is worth exploring a potential effect of commercial kits from different manufacturers on the test of non-fasting blood lipids in the further study.

In conclusion, LDL-C level decreased significantly after three daily meals in Chinese patients at high or very high risk of ASCVD, especially when TG level reached its peak after lunch. Relatively, non-HDL-C level was more stable than LDL-C level postprandially. Therefore, when LDL-C level was measured in the non-fasting state, non-HDL-C level could be evaluated simultaneously to reduce the interference of related factors, such as postprandial hypertriglyceridemia, on detection.

## Data Availability Statement

The raw data supporting the conclusions of this article will be made available by the authors, without undue reservation.

## Ethics Statement

The studies involving human participants were reviewed and approved by the Ethics Committee of the Second Xiangya Hospital of Central South University. The patients/participants provided their written informed consent to participate in this study.

## Author Contributions

LL and TW designed the study. YT, QL, JX, LZ, YX, and LG participated in the data collection. YT analyzed the data and prepared the figures and tables. YT and LL wrote the manuscript. LL revised the manuscript. All authors approved the final manuscript.

## Conflict of Interest

The authors declare that the research was conducted in the absence of any commercial or financial relationships that could be construed as a potential conflict of interest.

## Publisher’s Note

All claims expressed in this article are solely those of the authors and do not necessarily represent those of their affiliated organizations, or those of the publisher, the editors and the reviewers. Any product that may be evaluated in this article, or claim that may be made by its manufacturer, is not guaranteed or endorsed by the publisher.

## Abbreviations

ASCVD: atherosclerotic cardiovascular disease
TC: total cholesterol
HDL-C: high-density lipoprotein cholesterol
Non-HDL-C: non-high-density lipoprotein cholesterol
LDL-C: low-density lipoprotein cholesterol
TG: triglyceride
RC: remnant cholesterol
TRLs: triglyceride-rich lipoproteins
CHD: coronary heart disease
HBP: hypertension
BMI: body mass index
DM: diabetes mellitus
SBP: systolic blood pressure
DBP: diastolic blood pressure
CETP: cholesteryl ester transfer protein
NMR: nuclear magnetic resonance
smLDL: small and dense LDL.
