# Supplementary material for: Non-fasting Changes in Blood Lipids After Three Daily Meals Within a Day in Chinese Inpatients With Cardiovascular Diseases
Source: Front Cardiovasc Med. 2022 Apr 12;9:799300. doi: 10.3389/fcvm.2022.799300 (PMC9039513; doi:10.3389/fcvm.2022.799300)
Supplement: Supplementary file 1 [file Table_1.docx]

**Supplement Table 1:** Absolute changes in non-fasting blood lipid levels at 4 h after three meals and increased/decreased cases in two groups.

| Analyte(Units) | Fasting | 4h after breakfast | 4 h after lunch | 4 h after supper |
| --- | --- | --- | --- | --- |
| TC(mmol/L)-Male | 4.03±0.85 | 3.87±0.80^*$^ | 3.94±0.77 | 3.73±0.79^***&^ |
| Absolute change(mmol/L) | / | -0.15±0.45^&$^ | -0.09±0.40 | -0.30±0.43^&^ |
| Percent change(%) | / | -3.1±10.7^&$^ | -1.4±9.9 | -6.9±10.1^&^ |
| Increased/decreased | / | 18/28 | 17/29 | 10/38 |
| TC(mmol/L)-Female | 4.06±1.00 | 3.97±0.96^$^ | 3.99±0.92 | 3.84±0.87^&^ |
| Absolute change(mmol/L) | / | -0.09±0.56^$^ | -0.07±0.54 | -0.22±0.63^&^ |
| Percent change(%) | / | -1.00±14.3^$^ | -0.40±15.2 | -3.80±16.9^&^ |
| Increased/decreased | / | 14/15 | 13/16 | 12/16 |
| HDL-C(mmol/L)-Male | 1.00±0.22^#^ | 1.00±0.23^##^ | 1.03±0.21^##^ | 1.03±0.21^##^ |
| Absolute change (mmol/L) | / | 0.00±0.11 | 0.03±0.12 | 0.02±0.11 |
| Percent change(%) | / | 0.3±11.5 | 3.5±12.2 | 2.9±10.5 |
| Increased/decreased | / | 26/20 | 30/18 | 33/15 |
| HDL-C(mmol/L)-Female | 1.20±0.31 | 1.16±0.28 | 1.19±0.27 | 1.17±0.24 |
| Absolute change (mmol/L) | / | -0.04±0.16 | -0.01±0.14 | -0.03±0.16 |
| Percent change(%) | / | -2.03±13.6 | 0.67±13.8 | 0.22±14.6 |
| Increased/decreased | / | 12/17 | 15/14 | 14/14 |
| Non-HDL-C(mmol/L)-Male | 3.02±0.82 | 2.87±0.78^*$^ | 2.91±0.79 | 2.70±0.77^***&^ |
| Absolute change (mmol/L) | / | -0.15±0.42^$^ | -0.12±0.41 | -0.32±0.41^&^ |
| Percent change(%) | / | -4.1±13.4^$^ | -2.9±13.4 | -10.1±13.0^&^ |
| Increased/decreased | / | 16/32 | 18/28 | 8/40 |
| Non-HDL-C(mmol/L)-Female | 2.90±0.93 | 2.82±0.88^$^ | 2.80±0.90 | 2.67±0.83^*&^ |
| Absolute change (mmol/L) | / | -0.08±0.46^$^ | -0.10±0.48 | -0.23±0.55^&^ |
| Percent change(%) | / | -1.54±16.9^$^ | -1.95±18.5 | -5.96±20.7^&^ |
| Increased/decreased | / | 12/16 | 11/17 | 8/20 |
| LDL-C(mmol/L)-Male | 2.56±0.71 | 2.08±0.65^**&^ | 1.98±0.56^***^ | 2.01±0.62^***^ |
| Absolute change (mmol/L) | / | -0.47±0.49^&^ | -0.58±0.53 | -0.55±0.44 |
| Percent change(%) | / | -17.3±17.5^&^ | -20.9±16.1 | -20.8±15.4 |
| Increased/decreased | / | 4/43 | 4/44 | 5/43 |
| LDL-C(mmol/L)-Female | 2.49±0.83 | 2.11±0.73^***&^ | 1.99±0.63^***^ | 1.97±0.65^***^ |
| Absolute change (mmol/L) | / | -0.38±0.53 | -0.50±0.60 | -0.52±0.61 |
| Percent change(%) | / | -13.6±20.2^&$^ | -18.0±21.1 | -18.6±20.6 |
| Increased/decreased | / | 7/21 | 3/26 | 2/27 |
| TG(mmol/L)-Male | 1.84±1.21 | 2.49±1.51^***&^ | 2.81±1.63^***^ | 2.30±1.42^**&^ |
| Absolute change (mmol/L) | / | 0.65±1.21^&^ | 0.97±1.26 | 0.46±0.85^&^ |
| Percent change(%) | / | 44.1±54.2^&^ | 70.9±88.9 | 31.1±51.5^&^ |
| Increased/decreased | / | 40/8 | 39/9 | 31/17 |
| TG(mmol/L)-Female | 1.42±0.80 | 2.16±1.29^***^ | 2.37±1.41^***^ | 2.07±1.18^**^ |
| Absolute change (mmol/L) | / | 0.75±0.82^&$^ | 0.95±0.95 | 0.65±1.03 |
| Percent change(%) | / | 62.1±56.0 | 79.1±69.7 | 55.5±65.5 |
| Increased/decreased | / | 23/6 | 27/2 | 24/5 |
| RC (mmol/L)-Male | 0.46±0.20 | 0.79±0.48^***&^ | 0.93±0.61^***^ | 0.69±0.49^***&^ |
| Absolute change (mmol/L) | / | 0.32±0.46^&^ | 0.47±0.52 | 0.23±0.39^&^ |
| Percent change(%) | / | 74.8±88.2^&$^ | 101.7±99.8 | 49.5±87.3^&^ |
| Increased/decreased | / | 42/6 | 45/2 | 35/11 |
| RC (mmol/L)-Female | 0.41±0.26 | 0.70±0.39^***&^ | 0.81±0.51^***^ | 0.70±0.40^***^ |
| Absolute change (mmol/L) | / | 0.29±0.42^&^ | 0.40±0.52 | 0.29±0.43 |
| Percent change(%) | / | 88.6±94.9^&^ | 119.1±125.3 | 93.9±127.1 |
| Increased/decreased | / | 26/3 | 26/3 | 26/3 |

Note: Change (i.e. Absolute change) in blood lipid levels were calculated by the formula, i.e. each non-fasting level minus fasting level. Increased cases referred to patients whose non-fasting blood lipid levels were higher than the fasting levels, while decreased cases referred to those whose non-fasting blood lipid levels were lower than the fasting levels.

^*^*P* < 0.05, ^**^*P* < 0.01 and ^***^*P* < 0.001 when compared with the fasting level in the same group.

^$^*P* < 0.05 when compared with the level at 4 h after supper in the same group.

^&^*P* < 0.05 when compared with the level at 4 h after lunch in the same group.

^#^*P* < 0.05, ^##^*P* < 0.01 when compared with the female group at the same time-point.

Cholesterol: 1 mmol/L = 38.7 mg/dL. TG: 1 mmol/L = 88.6 mg/dL.
